# A sustainable approach for the stability study of psychotropic substances using vitreous humor and liver as alternative matrices

**DOI:** 10.1007/s00216-022-04064-w

**Published:** 2022-05-05

**Authors:** Anna Wójtowicz, Marcin Reciak, Paweł Mateusz Nowak, Renata Wietecha-Posłuszny

**Affiliations:** Laboratory for Forensic Chemistry, Department of Analytical Chemistry, Faculty of Chemistry, Jagiellonian University, 2 Gronostajowa St., 30-387 Kraków, Poland

**Keywords:** SPME/LC-MS, Postmortem analysis, Vitreous humor, Liver, Green analytical chemistry, RGB model

## Abstract

The stability of psychotropic substances representing various drug groups important from the perspective of forensic chemistry, including benzodiazepines, antidepressants, carbamazepine, cocaine, and their selected metabolites, was investigated for 1 month in two alternative biological matrices, vitreous humor and liver homogenate. Three different thermal storage conditions (−20, 4, and 20 °C) were tested. Liquid chromatography-mass spectrometry (LC-MS) analysis was preceded by an effective solid-phase microextraction (SPME) procedure. The results were statistically analyzed using one-way ANOVA to find significant concentration variations over time. The results obtained allowed for dividing the analytes into four groups: stable under all tested conditions, only at −20 and 4 °C, only at 20 °C, and overall unstable. Nordiazepam, venlafaxine, and cocaine and its metabolites turned out to be the most unstable substances, while fluoxetine showed the highest storage stability in both matrices. The SPME/LC-MS method was comprehensively evaluated according to the principles of white analytical chemistry (WAC), which reconcile the greenness and functionality of the method. A close to 100% whiteness score proves its sustainability and suitability for the intended application.

**Graphical abstract:**

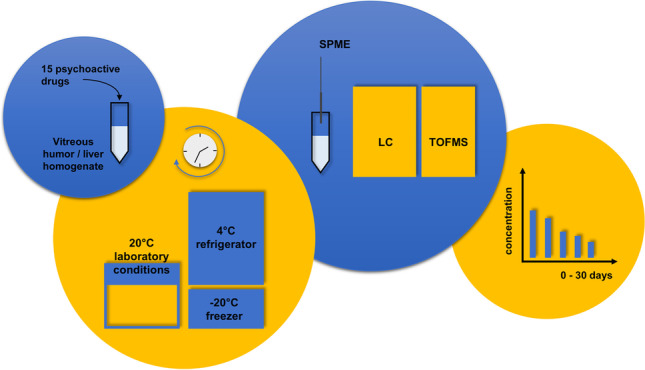

**Supplementary Information:**

The online version contains supplementary material available at 10.1007/s00216-022-04064-w.

## Introduction

In forensic toxicology, it is very important to correctly determine the concentration of primary xenobiotics as well as their metabolites in the biological matrices available in a given case, that is, both classical matrices, such as blood, and alternative ones, such as vitreous humor or liver. The result of such analysis may play an important role in determining how the crime occurred, finding the suspect guilty, or, in the event of a fatal event, determining the cause of death [1]. However, the analysis is hampered by the fact that the concentration of xenobiotics can change over time after death, mainly due to the postmortem redistribution [2] or degradation of the analyte in the samples [1]. Therefore, for cadavers that have decomposed under different conditions for different periods of time or in cases where autopsy samples were analyzed for up to several days, the results of the toxicological analysis may differ significantly from the concentration of the analytes at the time of death [3]. Thus, it is important to investigate the stability of various analytes in biological matrices during storage under different conditions. Additionally, the subject of stability studies should be the widest possible group of compounds of various structures to obtain the broadest possible information on the potential significance of this effect (stability) for laboratory practice.

Blood is the most widely used biological matrix in forensics. It can be easily obtained from humans, and thus many protocols for its analysis have been developed. However, it is susceptible to microbial growth and may undergo autolysis or putrefaction, so in some cases, such as in advanced decomposition of the cadaver, it may not be available [4]. In addition, there are reports of postmortem redistribution of xenobiotics in blood. Barnhart et al. [5] showed that the postmortem concentrations of methamphetamine and amphetamine were higher in the cardiac blood than in the heart muscle. Therefore, alternative matrices are sought.

One frequently used alternative biological matrix is vitreous humor (VH). It is a gel-like tissue located in the posterior chamber of the eye. Because of its location inside the eyeball and isolation from the rest of the body by the retina, VH is well protected against contamination and postmortem redistribution [6–8]. Our group recently reviewed the contemporary trends in VH drug analysis for toxicological and forensic purposes [9]. The VH matrix was found to be more stable than the blood matrix, especially for heroin metabolites [10] and gamma-hydroxybutyric acid (GHB) [11]. In addition, the stability of drugs in VH was reported to increase with a decrease in temperature and the addition of preservatives such as NaF or KF [12, 13].

Another important alternative biological matrix in forensic toxicology is the liver. The main functions of the liver are the metabolism of xenobiotics, the excretion of bile, the storage of vitamin reserves, and the production of various proteins, e.g., albumins. Because of these functions, the concentration of analytes in the liver is generally higher than that in blood, so the liver can be used successfully when blood is not available [14]. Regarding the stability of analytes in the liver, Kiszka et al. [15] found that the rate of cocaine decomposition in the liver is slower than that in the blood at the same temperature. This was explained by a greater decrease in enzyme activity in the liver, which may be related to the influence of pH. Since liver tissue contains more compounds that can cause acidity than blood, the reduction in enzyme activity due to the decrease in pH will be greater in this case [15].

Psychotropic substances (PS) most commonly found in biological matrixes in forensic cases include drugs from the groups of benzodiazepines [16–19], tricyclic antidepressants (TCAs) [16, 20, 21], selective serotonin reuptake inhibitors (SSRIs) [16, 21] and serotonin and norepinephrine reuptake inhibitors (SNRIs) [16, 21], carbamazepine [22], and numerous illicit drugs such as cocaine, opioids, and cannabis [23, 24]. A characteristic feature of all these substances is their psychoactive effect. Due to the metabolism of substances introduced into the body, it is also very important to detect the relevant metabolites in the examined tissues and organs to determine the actual concentration of the primary substances.

Reports on the stability of PS in alternative biological matrices are still limited, usually focusing on single groups of analytes [15, 17, 24] and/or relating to storage only at low temperatures [13]. The stability of benzodiazepines was studied in postmortem blood, bile, and VH samples stored at different temperatures over 6 months. The benzodiazepine concentration was shown to remain almost unchanged in all samples stored at −20 and −80 °C. Furthermore, some benzodiazepines were relatively stable for several weeks at 4 and 25 °C [17]. Another stability study by Holmgren et al. [13], in addition to benzodiazepines, included drugs from the group of antidepressants, analgesics, and hypnotics, and was carried out on postmortem femoral blood and VH stored for 1 year at −20 °C. Concentration changes were observed only for seven substances: a decrease in the concentration of ethanol, desmethylmianserin, 7-amino-nitrazepam, THC, and zopiclone, and an increase in the concentration of ketobemidone and thioridazine. Regarding illicit drugs, cocaine in VH was studied by Peres et al. [24] in terms of short-term stability (room temperature, 24 h), freeze-thaw stability (three cycles), and long-term stability (−20 °C, 7 and 30 days). VH samples were collected in vials containing 2% NaF. No degradation of the analyte was observed under any of the tested conditions. Kiszka et al. [15] also studied the stability of cocaine, but in four biological matrices of blood, liver, kidney, and brain and at three temperatures of 25, 4, and −20 °C for 90 days. The study showed that freezing tissues to −20 °C ensured the stability of cocaine practically throughout the duration of the experiment. In the case of liver samples stored at 25 °C, approximately 70% of the cocaine was degraded after 7 days. A slightly slower degradation rate was observed at 4 °C: after 1 day, a reduction by approximately one fourth of the initial concentration, an average loss of 48% after 7 days, and 62% reduction in the first month of storage [15].

As can be seen from the above examples, the stability of the concentration of psychoactive drugs in alternative biological matrices is not fully understood. In light of the importance of the results of quantitative analysis in determining the cause of death, analytically proven methods are needed for this purpose, including an effective stage of sample preparation and the simultaneous determination of analytes with high sensitivity and precision using a high-resolution separation technique. Other criteria are also important to consider, such as practicality and compliance with the idea of ​​green analytical chemistry (GAC). Therefore, this work focused on examining the stability of the most popular and most important—from the perspective of forensic chemistry—psychotropic drugs, and their few metabolites in two alternative matrices: VH and liver homogenate (LH). Animal biological samples were applied to use matrices with a confirmed lack of the tested analytes, blank matrices to which drugs were added only at the stage of sample preparation. In light of the complexity of the matrix and the need to simultaneously analyze many structurally diverse compounds, we used a previously developed and verified method based on solid-phase microextraction (SPME) in combination with liquid chromatography-mass spectrometry (LC-MS) applied to blood samples. The quality of this approach compared to the available alternatives was already demonstrated in our previous work [25]. The current method was subjected to some modifications taking into account the specifics of the biological matrices used as an alternative to blood, and then validated and re-evaluated. We applied the previously proposed 12 principles of white analytical chemistry (WAC), which are an extension of the known 12 principles of GAC. Besides greenness, WAC includes “red” and “blue” criteria corresponding to the analytical and practical functionality of the method [25]. The results showed that the applied method is fully sustainable, i.e., reasonably green and fit for purpose in terms of other important functional features.

## Materials and methods

### Chemicals and reagents

Standard solutions of the analytes alprazolam (ALP), citalopram (CIT), diazepam (DIAZ), doxepin (DOX), flunitrazepam (FLUN), fluoxetine (FLUOX), carbamazepine (CARB), cocaethylene (COCET), cocaine (COC), norcocaine (NORCOC), nordiazepam (NORDIAZ), nortriptyline (NORTR), oxazepam (OXA), temazepam (TEM), and venlafaxine (VEN) were purchased from Lipomed AG (Arlesheim, Switzerland). Deuterated derivatives of the analytes alprazolam-d5, diazepam-d5, flunitrazepam-d3, fluoxetine-d6, carbamazepine-d10, nordiazepam-d5, oxazepam-d5, and temazepam-d5, used as internal standards, were also purchased from Lipomed AG (Arlesheim, Switzerland). The selected number of 15 analytes, including six metabolites, was considered sufficient given that it was a preliminary study, and increasing the complexity of the method could potentially cause selectivity problems.

High-purity LC-MS chromatographic solvents acetonitrile, methanol, isopropyl alcohol, and sodium hydroxide obtained from Fluka Analytical (Seelze, Germany) were used in the experiment. Analytical-grade solvents acetonitrile, methanol, isopropyl alcohol, sodium hydroxide, and ammonium formate from Sigma-Aldrich (St. Louis, MO, USA) and formic acid obtained from Merck (Darmstadt, Germany) were also used. Deionized ultrapure water (18.2 M Ω · cm, TOC < 5 ppb) was obtained with the Milli-Q Plus system (Millipore, Bedford, MA, USA).

### LC-MS analysis

LC-MS analysis was performed using the UltiMate 3000 RS ultrahigh-performance liquid chromatography system (UHPLC; Dionex, Sunnyvale, CA, USA) equipped with a Hypersil GOLD Phenyl column (50 mm × 2.1 mm I.D., particles 1.9 μm: Thermo Scientific, Bremen, Germany). The chromatograph was coupled to a mass spectrometer with an electrospray ionization source and a MicrOTOF-Q II time-of-flight analyzer (Bruker, Bremen, Germany). The detection was based on the intensity analysis of the [M+H]^+^ ion signals in the range of approximately 237 to 337 m/z and the retention times of the analytes. The software used in the LC-MS analysis included Chromeleon 6.8 (Dionex) and software provided by Bruker Daltonics: HyStar 3.2, Data Analysis 4.0, QuantAnalysis 2.0, and Compass IsotopePattern.

The parameters of the LC-MS method were selected on the basis of the previous study by our laboratory [26]. Two mobile phases were used. Phase A contained 0.1% formic acid in ultrapure water and phase B was acetonitrile. The phases were pumped at a flow rate of 0.3 mL/min at 35 °C. The gradient elution program was used as follows: phase B: 0 min, 15%; 4–7 min, 40%; 10 min, 70%; 12.5–17 min, 15%. Three injections were used for each sample. For the MS parameters, positive ionization mode, capillary voltage of 4.5 kV, nebulizer pressure of 2.5 bar, dry gas flow of 5.5 L/min, dry gas temperature of 200 °C, and mass range of 50–800 m/z were applied. Before each sequence, the mass calibration of the cluster was performed using a 10 mM sodium formate solution in a mixture of isopropanol and water (1:1, v/v). Additionally, the calibration solution was supplied to the MS throughout the entire run using a valve, allowing the calibration solution to be injected before each measurement.

### Sample preparation

#### Biological samples

Postmortem bovine VH was collected from the eyes of calves provided by a meat plant (Laskopol, Poland). The VH samples were then centrifuged (14,000 rpm, 10 min) using an Allegra X-30R centrifuge (Beckman Coulter, Brea, CA, USA). The liver was purchased from a grocery store and homogenized using a TEquipment SCILOGEX D160 homogenizer (Boston, MA, USA) with the addition of a saline solution (0.9% NaCl) in a 1:3 ratio for 1 min. The supernatant was transferred to falcons and centrifuged (14,000 rpm, 10 min).

#### Analyte standard solutions

Mixtures of PS and their deuterated derivatives (internal standards, IS) were prepared from working standard solutions in methanol at a concentration of 10 μg/mL. The concentration of analytes in the PS and IS mixtures was 250 ng/mL for each compound. The PS mixture contained a total of 15 analytes and the IS mixture contained eight analytes, as not all deuterated derivatives were available.

#### Biological samples containing analytes for stability studies

For the preparation of biological samples containing analytes for stability studies, the appropriate amounts of the PS mixture were evaporated in a concentrator (20 min, 45 °C); then VH/LH was added and the samples were stored until analysis under various conditions: at room temperature (20 °C), in the refrigerator (4 °C), and in the freezer (−20 °C). The time points selected for the stability studies included 0, 1, 3, 6, 12, 18, and 30 days prior to measurement. One sample was prepared for each time point and conditions tested. On the day of analysis, the appropriate amounts of the IS mixture were evaporated, then the previously prepared biological sample containing PS was added and mixed. Subsequently, SPME extraction was performed. Two independent SPME extractions were performed from each sample; two 200 μL aliquots of each solution were collected. The concentration of PS and IS in each sample was constant and equal to 50 ng/mL.

The samples for the calibration curves were prepared on the day of measurement similarly to those for the stability studies. PS concentrations in the samples used to prepare the calibration curves were 2.5, 5, 10, 30, 50, 75, and 100 ng/mL, while the IS concentration was kept constant at 50 ng/mL. The samples for the calibration curves were analyzed by taking only one portion of each solution for the SPME procedure.

Because this was a preliminary study, only one analyte concentration was tested, and, for comparison purposes, it was the same for both biological matrices studied. Due to the expected lower concentrations of the tested drugs, especially in the VH, a concentration of 50 ng/mL, in the middle of the calibration curve (2.5–100 ng/mL), was selected for the stability study.

### SPME procedure

SPME-LC fiber probes (45 μm C18-Silica; Merck, Darmstadt, Germany) were used for the microextraction. The SPME procedure was derived from a previously published study [26]. The extraction was performed manually. The first step included conditioning of the SPME fibers. The fibers were placed directly in vials containing a mixture of methanol and water (1:1, v/v) and then shaken for 45 min at 2200 rpm. In the next step, 200 μL of biological samples (for the stability studies or calibration curve) was pipetted into inserts, which were placed in vials, and then the SPME fibers were introduced. The vials were then shaken for 60 min to adsorb the analytes on the fibers. After the adsorption process, the fibers were cleaned by immersing them in distilled water and shaking for approximately 10 s on a vortex to remove any residual biological matrix. A mixture of acetonitrile, methanol, and 0.1% formic acid (2:2:1. v/v/v) was used for desorption; the desorption time was 30 min. The solution obtained after desorption was evaporated in a concentrator (20 min, 45 °C) and then the mobile phase (50 μL of 0.1% formic acid) was added. The samples were then subjected to the LC-MS analysis. At the same time, the SPME fibers were cleaned to allow reuse in subsequent analyses. For this, they were shaken for 60 min in a solution of methanol/water/isopropanol (2:2:1, v/v/v).

## Results and discussion

### SPME/LC-MS analysis

#### Calibration

Analyte concentrations in samples stored at different temperatures were determined on the basis of the respective calibration plots. Calibration curves were prepared by plotting the ratio of the area under the peak for a given PS to the area under the peak of the corresponding internal standard (*y*-axis) as a function of the standard concentration (*x*-axis). IS selected for the individual PS in VH and LH are presented in Online Resource 1.

Calibration plots included seven levels of PS concentration: 2.5, 5, 10, 30, 50, 75, and 100 ng/mL. The trend line was fitted to the results obtained and its equation was determined using the linear regression method in Microsoft Excel. For each analyte, the value of the regression coefficient (*R*^2^) was greater than 0.99 for VH and 0.98 for LH, which proved the satisfactory linearity of the calibration curves. Detailed data on the linearity of the respective substances can be found in Online Resource 2. Data on precision and accuracy determined at the concentration of 50 ng/mL selected for the stability studies are presented in Table 1.

**Table 1:** Precision and accuracy of the determination of psychotropic substances in VH and LH using the SPME/LC-MS method

Vitreous humor								
	ALP	CIT	DIAZ	DOX	FLUN	FLUOX	CARB	
Mean [ng/ml]	53.33	45.60	46.47	46.60	52.36	45.61	44.32	
SD [ng/mL]	11.89	3.14	1.87	4.24	5.09	8.90	2.14	
Precision CV [%]	22.29	6.90	4.02	9.10	9.73	19.52	4.82	
Accuracy [%]	106.66	91.19	92.93	93.21	104.72	91.21	88.64	
	COCET	COC	NORCOC	NORDIAZ	NORTR	OXA	TEM	VEN
Mean [ng/ml]	53.50	58.65	48.37	46.27	45.64	50.52	50.04	55.89
SD [ng/mL]	8.04	1.85	6.76	2.48	9.93	7.25	6.99	3.16
Precision CV [%]	15.02	3.15	13.97	5.37	21.75	14.36	13.96	5.65
Accuracy [%]	107.00	117.31	96.74	92.55	91.27	101.05	100.09	111.78
Liver homogenate								
	ALP	CIT	DIAZ	DOX	FLUN	FLUOX	CARB	
Mean [ng/ml]	53.22	49.66	53.90	44.48	44.01	48.32	56.51	
SD [ng/mL]	7.18	6.81	5.90	5.50	5.83	3.14	2.22	
Precision CV [%]	13.50	13.71	10.95	12.36	13.25	6.50	3.94	
Accuracy [%]	106.45	99.33	107.81	88.96	88.03	96.64	113.02	
	COCET	COC	NORCOC	NORDIAZ	NORTR	OXA	TEM	VEN
Mean [ng/ml]	49.14	51.76	45.01	49.72	53.16	45.81	48.08	55.42
SD [ng/mL]	9.89	7.54	8.88	3.36	12.34	4.37	3.95	7.34
Precision CV [%]	20.12	14.56	19.72	6.76	23.21	9.54	8.21	13.24
Accuracy [%]	98.28	103.52	90.03	99.43	106.31	91.62	96.16	110.84

CV – coefficient of variation, SD – standard deviation, ALP – alprazolam, CIT – citalopram, DIAZ – diazepam, DOX – doxepin, FLUN – flunitrazepam, FLUOX – fluoxetine, CARB – carbamazepine, COCET – cocaethylene, COC – cocaine, NORCOC – norcocaine, NORDIAZ – nordiazepam, NORTR – nortriptyline, OXA – oxazepam, TEM – temazepam, VEN – venlafaxine

### Method evaluation according to the principles of WAC

The WAC concept is based on the red-green-blue color representation model, which was first used to evaluate analytical methods in 2019 [27]. It is an extension of the concept of the “green” method by adding two additional primary colors: red, denoting analytical performance, and blue, denoting practical and economic efficiency. As a result, the final color of the method depends on the extent to which the method can be defined as red, green, and blue. At best, the method is white, which signifies its completeness. In our last paper, we presented the development of the RGB model by formulating 12 WAC principles, divided into four red, four green, and four blue principles. For that purpose, the previously known 12 GAC principles were reduced to the four most primary, independent green criteria. A detailed description of the assumptions of the WAC concept and individual principles is available in the original work [25].

**Fig. 1:**
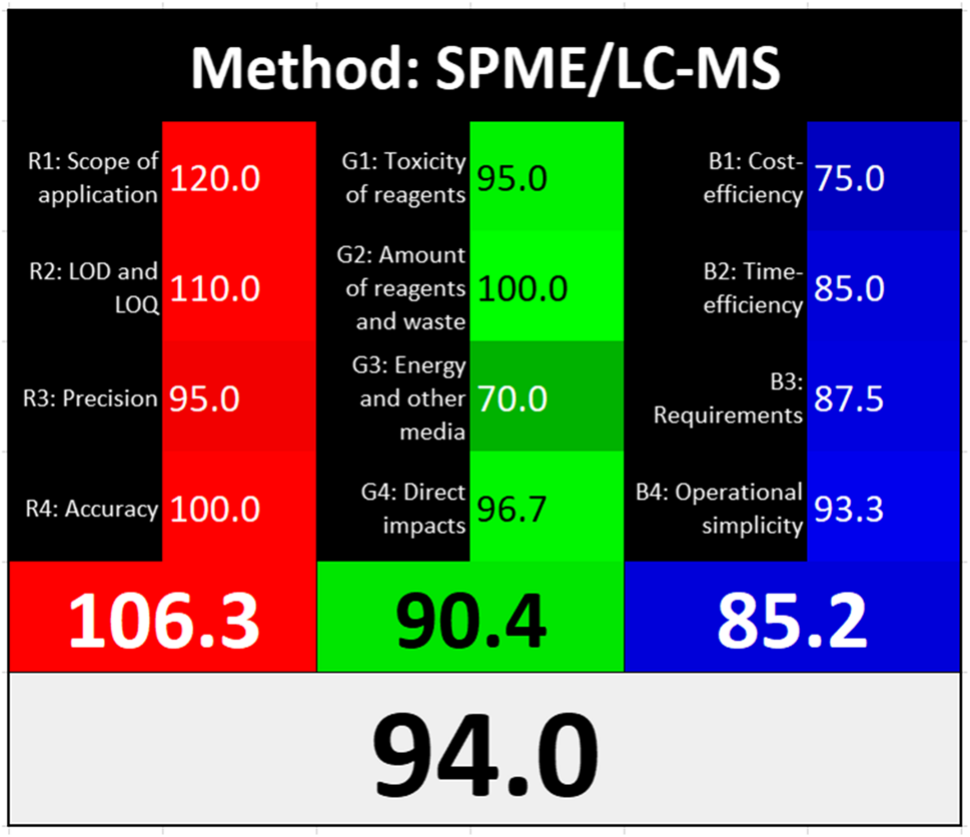
A summary of the assessment results obtained for the applied method according to the 12 principles of WAC (a detailed algorithm description is presented in the original work [25]). The values 106.3, 90.4, and 85.2 represent the average scores associated with the red, green, and blue criteria, respectively; 94.0 is the average total score including all 12 rules

As an example of using this model to compare many alternative methods, several different analytical methods for the determination of psychoactive compounds in biological material were tested [25]. The best overall result turned out to be SPME-LC/MS applied to blood samples, which was characterized by a high degree of balance of the red, green, and blue attributes. On this basis, this method was selected for the experiment described in this paper, subjecting it to the necessary modifications taking into account a larger number of analytes and a different type of matrix. This required the introduction of an additional centrifugation step during sample preparation. To determine to what extent the applied modifications influenced the overall degree of sustainability and overall effectiveness of the method, it was critically reassessed against the scores previously assigned to the individual 12 WAC principles for the original method. A summary of the method assessment results is illustrated in Fig. 1.

In the case of the red criteria, only the precision assessment was changed from 100 to 95, as upon validation the obtained relative standard deviation (RSD) values for the determination of analytes in VH and LH were slightly higher in total. In the case of the scope of application, the previous score was left unchanged, as the greater number of analytes increasing the usability of the method was compensated for by the lower linearity range. In the case of the limit of detection/limit of quantification (LOD/LOQ), the values improved; however, it should be borne in mind that in the case of VH and LH, taking into account the specificity of drug distribution and metabolism, lower concentrations should be expected, so at the same time, the requirements for LOD/LOQ are increasing for this type of samples. Therefore, the score remained unchanged. The accuracy expressed by recovery was similar for both methods; therefore, the assessment also remained unchanged.

For the green criteria, the toxicity of the reagents and the total amount of waste were evaluated in the same manner for both methods. However, the assessment of energy consumption changed from 75 to 70, due to the use of an additional centrifugation step. At the same time, a smaller number of harmful factors were identified—the remaining noise (resulting from the loud sound of the mass spectrometer) and solvent vapors (the bottles with the mobile phase were not isolated from the operator)—while the potential hazard resulting from working with human blood disappeared. Taking into account the fact that neither animals nor genetically modified organisms (GMOs) were used, the final score of the principle of direct impacts increased from 95 to 96.7 (a detailed algorithm for calculating points was described in the original paper [25]).

When considering the blueness, i.e., the practical and economic criteria, all parameters deteriorated slightly. The new method turned out to be a bit more expensive (change from 80 to 75), less time-efficient due to the additional centrifugation step (change from 90 to 85), requiring more laboratory infrastructure (centrifuge; change from 90 to 87.5), and also characterized by slightly worse operational simplicity (automation, portability; change from 100 to 93.3).

As can be seen from Fig. 1, the average assessment score of the red criteria remained very high, above 100, clearly indicating that from an analytical point of view, the choice of this method for the present experiment is beyond any doubt. The greenness of the method is rated lower, but it still exceeds 90, and this is largely due to the energy consumption of the method associated with the selection of advanced research instruments, mainly the mass spectrometer. However, given the analytical benefits offered, this decision seems justified. The practical aspects were assessed overall above 85, which is still a good result, also stemming from the advanced instrumental techniques used, which seem adequate for this purpose. The final whiteness index of the method is high, 94%, which indicates unambiguous appropriateness of the method and its high degree of balance of opposing attributes. This proves its true sustainability. The objectivity of this statement is supported by the reference to the other methods evaluated in the previous work [25], against which the procedure discussed here still looks the best overall. The key in this respect is the choice of an effective extraction method, a high-resolution separation method, and a sensitive detector, which are also reasonably good in terms of eco-friendliness and practicality compared to alternative techniques [25].

### Concentration changes

#### Concentration versus storage day plots

**Fig. 2:**
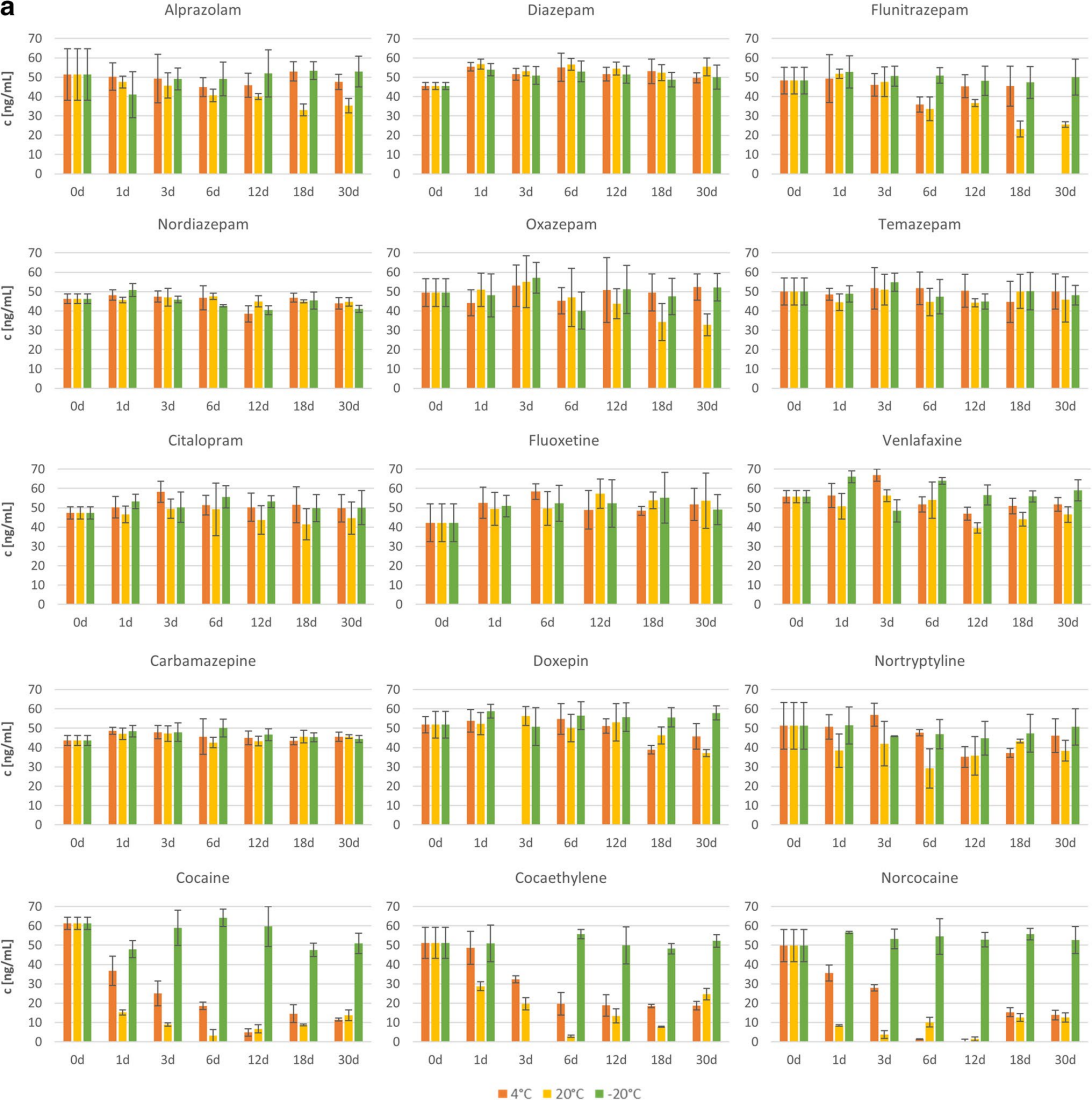

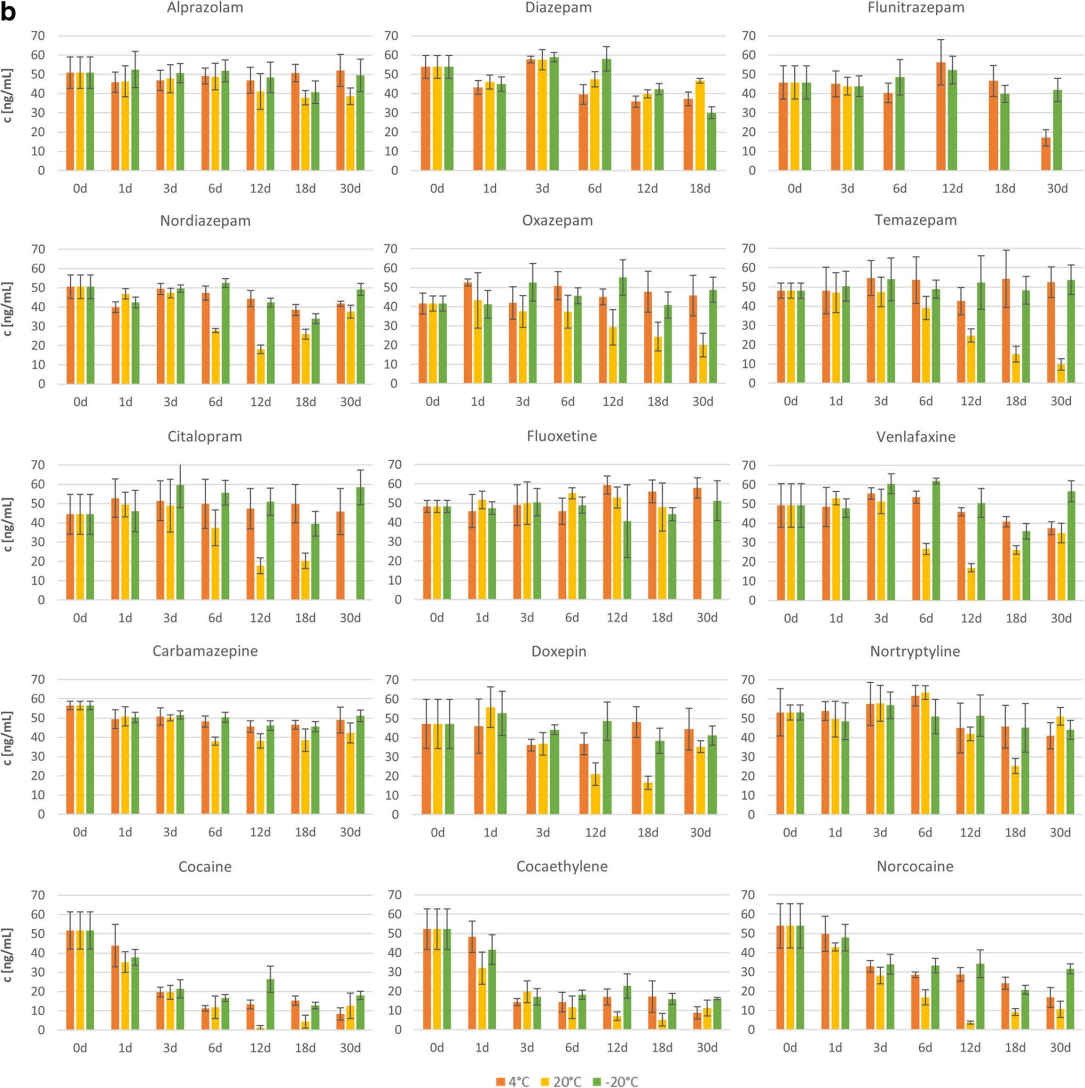
**a** Changes in the concentrations of individual psychotropic substances in VH on consecutive days of storage for 1 month at the three tested temperatures. **b** Changes in the concentrations of individual psychotropic substances in LH on consecutive days of storage for 1 month at the three tested temperatures

The values determined for the concentrations of individual PS in the consecutive days of storage, including the error value as +/− standard deviation, are graphically presented in Figs 2a and b for VH and LH, respectively. Each bar represents the concentration of the respective analyte obtained at a given temperature after the specified storage time (in days). It should be noted that some results were rejected and thus not included in the graphs due to strongly deviating concentration values. This was the case for FLUOX on day 3, COCET on day 3 at −20 °C, DOX on day 3 at 4 °C, and FLUN on day 30 at 4 °C in VH, and for CIT on day 30 at 20 °C, FLUOX on day 30 at 20 °C, FLUN from 6 to 30 days at 20 °C, and DIAZ on day 30 and DOX on day 6 in LH. The rejected results were characterized by concentration values that clearly exceeded the assumed value of 50 ng/mL, which could have resulted from some random errors in the sample preparation stage or could be related to errors in the internal standard method.

In general, it can be observed that during 1 month of storage, a temperature of −20 °C ensured the stability of most PS in VH. However, storage at 4 and 20 °C resulted in a significant drop in most PS concentrations, which was the fastest and led to the largest decrease in concentration after 1 month for COC and its two metabolites, NORCOC and COCET.

Based on the results obtained for LH, it can first be seen that fewer PS than in VH were stable when stored even at the lowest temperature of −20 °C. After only 24 h, a significant decrease in the concentration of COC and its metabolites was observed at all storage temperatures tested. A clear decrease in concentration after 12 days was also observed for DIAZ, NORDIAZ, and VEN. Higher storage temperatures, as in VH, further reduced the stability of PS in this matrix, such as CIT, OXA, and TEM, for which the concentration dropped significantly at 20 °C.

To evaluate the statistical significance of the observed concentration changes over time, a one-way analysis of variance (ANOVA) with temperature as a factor was performed in the next step.

#### One-way ANOVA

A one-way ANOVA was performed on the basis of the concentrations of individual analytes on consecutive days of storage at a given temperature, selecting the day of storage as a qualitative factor. The null hypothesis tested was the following:

- *H*_0_: Storage day *does not affect* PS concentration.

Thus, the alternative hypothesis was:

- *H*_A_: Storage day *affects* PS concentration.

Therefore, it was checked whether the differences in the average PS concentration over time were statistically significant and the PS could be considered stable (*H*_0_ acceptance) or unstable (*H*_0_ rejection) under given conditions. The calculations were performed in Statistica software, where the values of the *F*-statistic (*F*-value) and probability (*p*-value) were determined. The adopted significance level was 0.05 (*α*). If the *F*-value was greater than the value read from the *F*-distribution table for *α* = 0.05 and a given number of degrees of freedom (*df* = *n − 1*, where *n* is the number of trials), or the *p*-value was less than 0.05, the null hypothesis was rejected in favor of accepting the alternative hypothesis at the adopted significance level of 95%.

The ANOVA results obtained for each PS in both biological matrices tested are summarized in Table 2. The ANOVA results for the concentration changes are discussed below separately for each analyte tested.

**Table 2:** One-way ANOVA results obtained for VH and LH

Storage temp. [°C]	Vitreous humor						Liver homogenate					
	SS	*df*	MS	*F*	*p*	*H*_0_ rejected Yes/No?	SS	*df*	MS	*F*	*P*	*H*_0_ rejected Yes/No?
Alprazolam												
20	1109.6	6	184.9	5.564	0.00073	Yes	1476.9	6	246.1	3.246	0.01212	Yes
4	265.8	6	44.3	0.6048	0.72392	No	412.8	6	68.8	1.254	0.30438	No
−20	156.1	6	26.0	0.1997	0.97386	No	981.5	6	163.6	2.327	0.05384	No
Carbamazepine												
20	100.5	6	16.8	1.994	0.10161	No	2217.0	6	369.5	23.432	0.00000	Yes
4	116.0	6	19.3	0.928	0.48892	No	494	6	82.3	4.789	0.00123	Yes
−20	185.6	6	30.9	2.935	0.02315	Yes	495.8	6	82.6	12.62	0.00000	Yes
Citalopram												
20	271.1	6	45.2	0.6657	0.67789	No	9061.5	5	1812	12.7368	0.00000	Yes
4	311.2	6	51.9	1.158	0.35843	No	280.6	6	46.8	0.1381	0.99012	No
−20	274.3	6	45.7	1.243	0.32698	No	1572.5	6	262.1	1.9557	0.10285	No
Cocaethylene												
20	6884.0	6	1147	116.164	0.00000	Yes	7652.3	6	1275	31.5382	0.00000	Yes
4	6059.9	6	1010	39.887	0.00000	Yes	6884.8	6	1147	6.8738	0.00010	Yes
−20	120.8	5	24.2	0.3237	0.89306	No	4592.8	6	765.5	23.0233	0.00000	Yes
Cocaine												
20	12063	6	2011	415.118	0.00000	Yes	10063	6	1677	48.035	0.00000	Yes
4	11172	6	1862	94.216	0.00000	Yes	8273.1	6	1379	40.2716	0.00000	Yes
−20	1278	6	213.0	5.729	0.00074	Yes	6164.9	6	1027	43.744	0.00000	Yes
Diazepam												
20	310.2	6	51.7	4.821	0.00255	Yes	927.4	5	185.5	12.447	0.00000	Yes
4	239.9	6	49.0	2.305	0.06315	No	2127.8	5	425.6	26.486	0.00000	Yes
−20	198.7	6	33.1	1.696	0.15752	No	3430.3	5	686.1	34.005	0.00000	Yes
Doxepin												
20	795.4	6	132.6	1.8069	0.14210	No	5622.4	5	1124	19.3364	0.00000	Yes
4	864.4	5	172.9	3.443	0.01988	Yes	675.1	5	135.0	1.5237	0.22320	No
−20	229.6	6	38.3	0.69	0.66001	No	647.5	5	129.5	1.6288	0.19407	No
Flunitrazepam												
20	4079.8	6	680.0	25.324	0.00000	Yes	-	-	-	-	-	-
4	715.9	5	143.2	1.9223	0.13129	No	6091.5	5	1218	19.671	0.00000	Yes
−20	66.1	6	11.0	0.209	0.97131	No	329.7	5	65.93	1.319	0.28585	No
Fluoxetine												
20	605.1	5	121.0	1.4172	0.25705	No	145.0	5	29.01	0.48	0.7876	No
4	336.3	5	67.3	1.1137	0.38616	No	848.0	6	141.3	2.865	0.02722	Yes
−20	326.1	5	65.2	0.6238	0.68303	No	148.0	6	24.6	0.1776	0.98070	No
Norcocaine												
20	8493.7	6	1416	118.467	0.00000	Yes	9676.2	6	1613	51.3513	0.00000	Yes
4	8578.9	6	1430	89.061	0.00000	Yes	5380.1	6	896.7	22.661	0.00000	Yes
−20	99.1	6	16.5	0.447	0.84011	No	3106.4	6	517.7	12.193	0.00000	Yes
Nordiazepam												
20	128.7	6	21.5	3.04	0.01906	Yes	5824.7	6	970.8	89.717	0.00000	Yes
4	409.3	6	68.2	5.652	0.00051	Yes	897.5	6	149.6	4.83	0.00123	Yes
−20	283.4	6	47.2	5.835	0.00065	Yes	1602.7	6	267.1	24.069	0.00000	Yes
Nortriptyline												
20	1283.8	6	214.0	2.323	0.06270	No	4359.1	6	726.5	4.5228	0.00363	Yes
4	1681.3	6	280.2	7.004	0.00021	Yes	1741.3	6	290.2	1.6817	0.15872	No
−20	97.6	6	16.3	0.1989	0.97324	No	502.5	6	83.8	0.408	0.86760	No
Oxazepam												
20	2139.1	6	356.5	2.9392	0.02299	Yes	3361.6	6	560.3	6.9056	0.00009	Yes
4	185.9	6	31.0	0.2759	0.94298	No	437.5	6	72.9	1.0889	0.39827	No
−20	302.5	6	50.4	0.57	0.74992	No	686.9	6	114.5	1.977	0.10902	No
Temazepam												
20	282.6	6	47.1	0.776	0.59540	No	8154.5	6	1359	38.401	0.00000	Yes
4	177.1	6	29.5	0.392	0.87756	No	607.9	6	101.3	0.9033	0.50603	No
−20	347.6	6	57.9	1.371	0.26328	No	165.4	6	27.6	0.3556	0.89925	No
Venlafaxine												
20	1380.9	6	230.1	8.039	0.00003	Yes	6568.1	6	1095	32.999	0.00000	Yes
4	915.1	6	152.5	9.812	0.00001	Yes	1435.3	6	239.2	5.516	0.00071	Yes
−20	864.7	6	144.1	7.72	0.00011	Yes	2244.1	6	374.0	7.97	0.00006	Yes

SS – sum of squares, *df* – degrees of freedom, MS – mean squares, *F* – *F*-value, *p* – probability value

“−” indicates the lack of results due to insufficient data

## ALP

No grounds were found to reject the null hypothesis for this analyte when stored at temperatures of −20 and 4 °C in either matrix. However, at a temperature of 20 °C, a decrease in concentration was observed after about 6 days. Therefore, ALP was found to be stable in VH and LH during 30 days of storage only at −20 and 4 °C.

## CARB

The results of the statistical analysis obtained for CARB in VH indicated the stability of this analyte when stored at 4 and 20 °C and its instability at −20 °C. The lack of stability at −20 °C is not compatible with the assumption that stability will increase with decreasing storage temperature. Furthermore, when analyzing the course of changes in the concentration of this analyte over time (Fig. 2a), it can be seen that the CARB content seems to remain at a similar level at all storage temperatures, and the rejection of the null hypothesis was probably due to the low concentration on day 0. Therefore, the one-way ANOVA for this analyte was repeated after rejecting the 0-day data, and the results obtained are summarized in Table 3. This time, the results did not indicate any grounds for rejecting the null hypothesis in any of the cases tested, so it was concluded that CARB is stable in VH during 30 days of storage under all conditions tested: at −20, 4 and 20 °C.

**Table 3:** One-way ANOVA results obtained after the rejection of the 0-day data for CARB and DIAZ in VH, after the rejection of the 18-day data for DOX in VH, and after the rejection of the 12-day data for FLUOX in LH

Storage temp. [°C]	SS	df	MS	*F*	*P*	*H*_0_ rejected Yes/No?
Carbamazepine – VH						
20	100.1	5	20.0	2.144	0.09467	No
4	37.4	5	7.5	0.316	0.89867	No
−20	106.5	5	21.3	1.876	0.13615	No
Diazepam – VH						
20	54.1	5	10.8	0.884	0.51109	No
4	89.5	5	17.9	0.906	0.49439	No
−20	92.7	5	18.6	0.84	0.53419	No
Doxepin – VH						
20	714.3	5	142.9	1.6874	0.18601	No
4	222.4	4	55.6	0.914	0.47825	No
−20	228.5	5	45.7	0.758	0.59061	No
Fluoxetine – LH						
20	137.4	4	34.3	0.5129	0.72709	No
4	419.0	5	83.8	1.551	0.21338	No
−20	141.9	5	28.4	0.677	0.64520	No

However, for CARB in LH, the ANOVA results indicated the instability of this analyte under all storage conditions. The decrease in drug concentration occurred after the first day of storage, intensified on day 6, and was most pronounced at the highest temperature of 20 °C. Consequently, CARB in LH was found to be unstable at all temperatures tested: −20, 4 and 20 °C.

## CIT

CIT in VH was found to be stable under all storage conditions tested, but in LH only at temperatures of −20 and 4 °C. A decrease in the concentration of CIT in LH at 20 °C was observed from about the sixth day of storage.

## COC and its metabolites COCET and NORCOC

The ANOVA results showed that COC and its two tested metabolites were generally unstable in VH and LH, except for COCET and NORCOC, stable in VH at −20 °C.

For the VH matrix, a clear decrease in the concentration of COC and its two metabolites was observed after the first day of storage at 4 and 20 °C (Fig. 2a). The initial decrease in concentration was much faster at the temperature of 20 than at 4 °C, but then the concentration values have leveled out. It should also be mentioned that in the last days of storage, an increase in the concentrations of analytes was observed at these temperatures, which may be due to some random errors or may indicate the occurrence of more complicated processes. To investigate this phenomenon, further studies are needed for samples stored longer than 30 days. In contrast, for storage at −20 °C, a concentration decrease was observed only for COC and occurred on about day 18, while the concentration of both COC metabolites remained constant throughout the 30-day study period.

When analyzing the concentration–time plots for LH (Fig. 2b), a clear decrease in the concentration of COC and its two metabolites can be seen after the first day of storage. The intensity of the drop in concentration is greater as the storage temperature increases.

## DIAZ and its metabolites NORDIAZ, OXA, and TEM

**Fig. 3:**
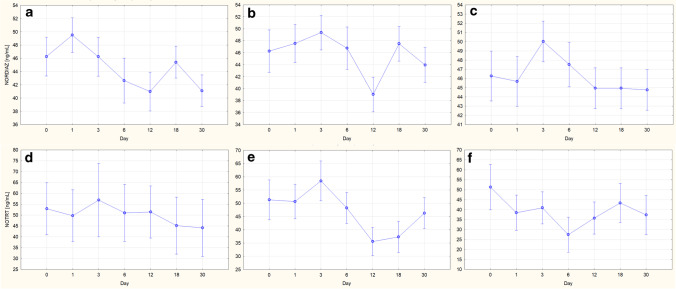
Changes in drug concentration in VH on consecutive days of storage: NORDIAZ at temperature of **a** −20, **b** 4, **c** 20 °C, and NORTR at temperature of **d** −20, **e** 4, **f** 20 °C. The vertical bars indicate 0.95 confidence intervals

The results of a one-way ANOVA performed for DIAZ and its metabolites in VH indicated the stability of DIAZ and OXA when stored at −20 and 4 °C, complete stability of TEM, and instability of NORDIAZ. These conclusions are consistent with the concentration changes over time shown in Fig. 2a for OXA and TEM, although questionable for DIAZ and NORDIAZ. In the case of DIAZ, as discussed above for CARB, the rejection of the null hypothesis at 20 °C may be due to a low concentration of the analyte in the sample on day 0. Therefore, the analysis was repeated after rejecting these data and the results obtained are presented in Table 3. The new results did not indicate any grounds for rejecting the null hypothesis at any temperature; therefore, DIAZ was considered stable in VH under all storage conditions tested. However, the case of NORDIAZ was more complicated. The results shown in Fig. 2a indicated similar concentration values, but the statistical analysis proved the significance of the observed differences. As shown in the concentration versus storage day plots (Fig. 3) obtained in Statistica, clear fluctuations in the concentration of the analyte were observed at each of the temperatures tested, which makes it difficult to unequivocally assess the storage stability of this analyte in VH. Even if removing the most distant outlier concentration values at 4 and 20 °C, so that the data obtained on days 12 and 3, respectively, would lead to the finding of no change in concentration over time for these storage conditions, elimination of the outliers for the data obtained at −20 °C would still indicate a declining trend in NORDIAZ concentration over time. The instability of this analyte in the sample stored at −20 °C, considered the most stable storage conditions, with stability at 4 and 20 °C, may indicate an error in this sample or difficulties in assessing the stability of this analyte by the SPME/LC-MS method used. Therefore, NORDIAZ in VH was found to be unstable under all the tested conditions.

The statistical results obtained for the LH samples showed instability of DIAZ and NORDIAZ under all storage conditions, and stability of OXA and TEM only at temperatures of −20 and 4 °C. These results agree with the changes observed in Fig. 2b. DIAZ and NORDIAZ concentrations decreased significantly on the sixth day of storage at 4 and 20 °C and on the 12th day at −20 °C. In the case of NORDIAZ, it is also worth noting the increase in the concentration of this analyte on the 30th day of storage under all tested conditions, which may indicate errors in these samples or may require further studies for longer storage periods. For OXA and TEM, the stability of the analyte concentration was observed at −20 and 4 °C, while at 20 °C the concentration decreased significantly after about 3–6 days.

## DOX

The ANOVA results obtained for DOX indicated the stability of this analyte in VH when stored at −20 and 20 °C, and in LH at −20 and 4 °C. Although the results obtained for LH are consistent with the assumption that stability increases with decreasing storage temperature, the results obtained for VH may indicate an error. By analyzing the changes in DOX concentration over time (Fig. 2a), it can be seen that the rejection of the null hypothesis for the results obtained at 4 °C was probably due to a randomly lower concentration value obtained on day 18. Therefore, the analysis was repeated after omitting the data for that day, and the results obtained are summarized in Table 3. The new results demonstrated the stability of DOX in VH during storage at all temperatures tested.

The conclusions drawn from the statistical analysis of changes in DOX concentration in LH correspond to the changes observed in Fig. 2b. At 20 °C, a clear decrease in concentration was observed on the 12th day of storage, and at −20 and 4 °C, no statistically significant changes were observed.

## FLUN

FLUN was found to be stable in VH when stored at −20 and 4 °C, but in LH only when stored at −20 °C. The decrease in FLUN concentration in VH at 20 °C began on day 6, while in LH at 4 °C, the decrease in concentration did not occur until day 30. It should also be noted that the ANOVA results could not be obtained for FLUN in LH stored at 20 °C due to the lack of data on the concentration values on days 6–30.

## FLUOX

The ANOVA results showed that FLUOX was stable in VH during storage at all temperatures tested, which is consistent with the data presented in Fig. 2a. For the LH matrix, instability in analyte concentration was only observed when stored at 4 °C. However, after analyzing the plot obtained for FLUOX concentration changes in Fig. 2b, it was concluded that the rejection of the null hypothesis, in this case, could have been caused by an incidentally high concentration value obtained on day 12. Therefore, the statistical analysis was repeated after discarding the results for that day. The results obtained, which are summarized in Table 3, indicated the stability of FLUOX also in the LH matrix during storage under all conditions tested.

## NORTR

The statistical analysis results obtained for NORTR in VH showed the stability of this analyte when stored at −20 and 20 °C. However, by analyzing the concentration versus storage day plots obtained in Statistica and shown in Fig. 3, it can be seen that the concentration of NORTR during storage at 20 °C decreased from day 0 to day 6 and then started to increase. The lack of stability of this analyte is clearly visible, and the acceptance of the null hypothesis was the total effect of the decrease and increase in concentration. It can therefore be concluded that NORTR in VH is stable when stored at −20 °C and unstable at 4 and 20 °C.

For NORTR in LH, the ANOVA results showed the stability of the analyte when stored at −20 and 4 °C. These results are consistent with the observed trend of concentration changes over time (Fig. 2b), including constant concentration values at −20 and 4 °C throughout the entire study, and a decrease in analyte concentration on day 12 when stored at 20 °C. At this temperature, attention should also be paid to the increase in concentration on day 30, which requires further investigation.

## VEN

For VEN, the results of the statistical analysis indicated the instability of the concentration during storage at all temperatures for both tested matrices. The decrease in concentration for the VH matrix was observed from day 12 and was highest at 20 °C, while for LH the decrease in concentration occurred at −20 and 4 °C also on day 12 and at 20 °C on day 6, and was also highest at this highest storage temperature. In the case of VEN in LH, an increase in concentration was also observed in the last days of storage, which was previously mentioned for the other drugs.

### Classification of the analytes depending on their stability

Based on the data presented in Tables 2 and 3 and Figs. 2 and 3, the analytes tested were divided into four groups that differed in stability as follows.

I Stable under all storage conditions
II Stable at 4 and −20 °C
III Stable at −20 °C
IV Unstable under all storage conditions.

The assignment of the analytes to the respective groups is presented in Table 4.

**Table 4:** Assignment of the analytes to appropriate stability groups

	Vitreous humor	Liver homogenate
I. Stable under all storage conditions	Carbamazepine Citalopram Diazepam Doxepin Fluoxetine Temazepam	Fluoxetine
II. Stable at 4 and −20 °C	Alprazolam Flunitrazepam Oxazepam	Alprazolam Citalopram Doxepin Nortriptyline Oxazepam Temazepam
III. Stable at −20 °C	Cocaethylene Norcocaine Nortriptyline	Flunitrazepam
IV. Unstable under all storage conditions	Cocaine Nordiazepam Venlafaxine	Carbamazepine Cocaethylene Cocaine Diazepam Norcocaine Nordiazepam Venlafaxine

FLUOX from the SSRI group was found to be the most stable analyte in both matrices under all conditions tested. The second SSRI drug, CIT, also showed good stability—under all conditions tested in VH and at −20 and 4 °C in LH. Benzodiazepines and TCAs were also relatively stable in both matrices during storage, except for NORDIAZ, which in both cases was unstable under all conditions tested, and DIAZ was unstable in LH. However, CARB was characterized by completely different stability for the two matrices tested, being stable under all conditions in VH and unstable under all conditions in LH. The lowest storage stability in both matrices, apart from the previously mentioned NORDIAZ, was found for VEN and COC and its metabolites. Therefore, special attention should be paid to the concentrations of these analytes when analyzing biological samples stored for some time, from a few days to 1 month.

The results obtained confirmed the previously reported good storage stability of PS at −20 °C [13, 18]. The instability of COC at higher temperatures (4 and 20 °C) was also confirmed, but in contrast to previous studies [15, 24], a decrease in COC concentration at −20 °C was observed after 18 days in VH and after only 24 h in LH. Differences in the stability of COC in VH may be due to the lack of added preservatives, whereas in the case of LH, stability was not tested in the whole organ, but only in the homogenate.

It is also worth noting that, considering the mean changes in concentration from all temperatures, the analyzed analytes can be divided into three groups: a group with relatively similar stability in both matrices, a group of drugs clearly more stable in VH than in LH, and a group clearly more stable in LH than in VH. The first includes ALP, CIT, FLUN, COCET, COC, NORCOC, NORTR, and OXA, the second (more stable in VH) DIAZ, CARB, NORDIAZ, TEM, and FLUOX, and the third (more stable in LH) DOX and VEN.

Attention should also be paid to the high values of the standard deviation of some results. It is suspected that this could have been affected by the SPME procedure, in particular by the multiple uses of SPME fibers. The tests carried out in our laboratory showed that the fiber cleaning process used and described in the manuscript is effective, and no fiber damage is observed after one series of experiments [26]. However, in the course of our study, the same group of fibers was used several times. Therefore, further research is needed on the multiple uses of SPME fibers.

### Hypothetical explanation for stability/instability

An attempt was made to explain the observed stability of the investigated psychotropic drugs taking into account their basic properties, such as molecular weight, hydrophobicity measured by logP value, topological polar surface area, water solubility, and acidity expressed by p*K*_a_. The relevant data were taken from the ChemBank open database. Attempts were made to compare the values of these parameters with the average changes in the concentrations of individual drugs to find a potential correlation. However, no significant trend was detected, indicating the lack of a direct link between the stability of the compounds under consideration and their basic physicochemical properties. Therefore, the most likely cause of the observed changes in concentrations is subtle structural differences that may affect supramolecular interactions of drugs with the cellular environment, including enzymes, carriers, and structural proteins, including the host organism as well as the bacterial, especially postmortem microflora. This hypothesis is supported by quite consistent results obtained for structurally similar compounds, e.g., COC and NORCOC. The specificity of a given organ also plays an important role, as evidenced by the significant differences between LH and VH. Therefore, further research on this topic seems necessary.

## Conclusions

The stability of structurally diverse PS from the group of benzodiazepines, tricyclic antidepressants, SSRIs, SNRIs, carbamazepine, cocaine, and their selected metabolites was studied in two biological matrices, LH and VH, using the SPME/LC-MS method. The study consisted in 30 days of storage at three different temperatures of −20, 4, and 20 °C. The results obtained allowed the classification of the analytes into four general groups: (I) stable at all tested temperatures, (II) only at 20 and 4 °C, (III) only at −20 °C, and (IV) generally unstable. COC, NORDIAZ, and VEN were found to be substances whose concentration changed significantly during storage and therefore should be carefully considered when determining them in biological samples. Additionally, in the LH matrix, CARB, COCET, DIAZ, and NORCOC also showed poor storage stability. FLUOX demonstrated the highest storage stability in both tested matrices under all tested conditions.

The results of the stability studies in the VH matrix showed that during 1 month of storage, a temperature of −20 °C prevents changes in the concentration of most of the substances tested. Twelve out of 15 analytes tested were found to be stable at this temperature. At the same time, only eight of the 15 analytes were stable at this temperature in the LH matrix. The number of storage-stable analytes at 4 and 20 °C was also greater in VH than in LH. At 4 °C, out of 15 tested analytes, nine were stable in VH and seven in LH. Similarly, at 20 °C, six analytes were stable in VH and only one in LH. The study conducted confirmed that VH is a biological matrix that ensures relatively high stability of the analytes and is therefore valuable in toxicological analysis.

We ensured the selection of the optimal method for this purpose based on the concept of ​​WAC, combining the evaluation of greenness with other criteria determining overall functionality. The applied procedure was based on a method originally used for blood samples, which was tested and evaluated in this respect in our previous work. Herein, it was subjected to the necessary modifications, validation, and critical reassessment using the same tool. In our opinion, the SPME/LC-MS used here deserves the name of “white” obtained from the evaluation outcomes, which in relation to the WAC concept means a high degree of balance between analytical performance (red), environmental friendliness (green), and practical efficiency (blue), which should also be understood as true evidence of sustainability.

## Supplementary Information

ESM 1 (DOCX 22 kb)

## Data Availability

Data and meta-data are available from the corresponding author at the request of the reader.

## Declarations

### Source of biological material

Animal samples were obtained from entities selling animal materials for consumer purposes and thus meet the requirements of the marketing authorization. The biological samples were taken postmortem. Laboratory animals were not used during the experiment and experiments were not carried out on live animals.
