# Supplementary material for: A sustainable approach for the stability study of psychotropic substances using vitreous humor and liver as alternative matrices
Source: Anal Bioanal Chem. 2022 May 5;414(21):6355–70. doi: 10.1007/s00216-022-04064-w (PMC9372124; doi:10.1007/s00216-022-04064-w)
Supplement: Supplementary file 1 — (DOCX 22 kb) [file 216_2022_4064_MOESM1_ESM.docx]

**Supplementary Information**

**A sustainable approach for the stability study of psychotropic substances using vitreous humor and liver as alternative matrices**

Anna Wójtowicz^1^, Marcin Reciak^1^, Paweł Mateusz Nowak^1^, Renata Wietecha-Posłuszny^1,*^

^1^ Laboratory for Forensic Chemistry, Department of Analytical Chemistry, Faculty of Chemistry, Jagiellonian University, Gronostajowa 2, 30-387 Kraków

* Corresponding author: Renata Wietecha-Posłuszny, Dr. habil.

Laboratory for Forensic Chemistry,

Department of Analytical Chemistry

Faculty of Chemistry, Jagiellonian University

2, Gronostajowa St., 30-387 Kraków, Poland

email: wietecha@chemia.uj.edu.pl

tel. +48 12 686 20 08 Fax. +48 12 686 27 50

**Online Resource 1** Assignment of internal standards for individual analytes

|  | Vitreous humor | Liver homogenate |
| --- | --- | --- |
| Analyte | Internal standard | Internal standard |
| alprazolam | alprazolam-d_5_ | alprazolam-d_5_ |
| carbamazepine | carbamazepine-d_10_ | carbamazepine-d_10_ |
| citalopram | diazepam-d_5_ | temazepam-d_5_ |
| cocaethylene | alprazolam-d_5_ | alprazolam-d_5_ |
| cocaine | alprazolam-d_5_ | alprazolam-d_5_ |
| diazepam | diazepam-d_5_ | diazepam-d_5_ |
| doxepin | diazepam-d_5_ | temazepam-d_5_ |
| flunitrazepam | flunitrazepam-d_3_ | flunitrazepam-d_3_ |
| fluoxetine | fluoxetine -d_6_ | flunitrazepam-d_3_ |
| norcocaine | diazepam-d_5_ | alprazolam-d_5_ |
| nordiazepam | nordiazepam-d_5_ | nordiazepam-d_5_ |
| nortriptyline | nordiazepam-d_5_ | nordiazepam-d_5_ |
| oxazepam | oxazepam-d_5_ | oxazepam-d_5_ |
| temazepam | temazepam-d_5_ | temazepam-d_5_ |
| venlafaxine | nordiazepam-d_5_ | nordiazepam-d_5_ |

**Online Resource 2** Data on linearity obtained for individual analytes

| Analyte | Slope [mL/ng] | Intercept | R^2^ | Range [ng/mL] | No. Replicas |
| --- | --- | --- | --- | --- | --- |
| Vitreous humor | | | | | |
| alprazolam | 0.0341 | -0.0902 | 0.9989 | 2.5 – 100 | 3 |
| carbamazepine | 0.0169 | -0.0184 | 0.9970 | 2.5 – 100 | 3 |
| citalopram | 0.0441 | 0.0341 | 0.9982 | 2.5 – 100 | 3 |
| cocaethylene | 0.0246 | -0.2156 | 0.9985 | 10 – 100 | 3 |
| cocaine | 0.0211 | 0.0368 | 0.9925 | 2.5 – 100 | 3 |
| diazepam | 0.0193 | -0.0180 | 0.9955 | 2.5 – 100 | 3 |
| doxepin | 0.0232 | -0.0577 | 0.9942 | 2.5 – 100 | 3 |
| flunitrazepam | 0.0060 | -0.0083 | 0.9993 | 5 – 100 | 3 |
| fluoxetine | 0.0207 | -0.0401 | 0.9969 | 2.5 – 100 | 3 |
| norcocaine | 0.0194 | 0.0511 | 0.9962 | 2.5 – 100 | 3 |
| nordiazepam | 0.0270 | -0.0565 | 0.9966 | 2.5 – 100 | 3 |
| nortriptyline | 0.0423 | 0.2404 | 0.9952 | 2.5 – 100 | 3 |
| oxazepam | 0.0170 | 0.0123 | 0.9956 | 2.5 – 100 | 3 |
| temazepam | 0.0365 | -0.1110 | 0.9961 | 2.5 – 100 | 3 |
| venlafaxine | 0.0175 | -0.0226 | 0.9990 | 2.5 – 100 | 3 |
| Liver homogenate | | | | | |
| alprazolam | 0.0256 | -0.0455 | 0.9847 | 2.5 – 100 | 3 |
| carbamazepine | 0.0144 | -0.0029 | 0.9952 | 2.5 – 100 | 3 |
| citalopram | 0.0374 | -0.0992 | 0.9916 | 2.5 – 100 | 3 |
| cocaethylene | 0.0481 | -1.1638 | 0.9836 | 30 – 100 | 3 |
| cocaine | 0.0181 | 0.1904 | 0.9831 | 2.5 – 100 | 3 |
| diazepam | 0.0208 | -0.0779 | 0.9856 | 2.5 – 100 | 3 |
| doxepin | 0.0301 | -0.2447 | 0.9919 | 10 – 100 | 3 |
| flunitrazepam | 0.0054 | 0.0062 | 0.9995 | 10 – 100 | 3 |
| fluoxetine | 0.0024 | -0.0071 | 0.9908 | 2.5 – 100 | 3 |
| norcocaine | 0.0399 | -0.7686 | 0.9917 | 50 – 100 | 3 |
| nordiazepam | 0.0239 | -0.0121 | 0.9999 | 2.5 – 100 | 3 |
| nortriptyline | 0.0212 | -0.0008 | 0.9908 | 2.5 – 100 | 3 |
| oxazepam | 0.0131 | 0.0217 | 0.9994 | 2.5 – 100 | 3 |
| temazepam | 0.0308 | -0.0648 | 0.9983 | 2.5 – 100 | 3 |
| venlafaxine | 0.0106 | -0.0908 | 0.9894 | 30 – 100 | 3 |
